# Ubiquinone Supplementation with 300 mg on Glycemic Control and Antioxidant Status in Athletes: A Randomized, Double-Blinded, Placebo-Controlled Trial

**DOI:** 10.3390/antiox9090823

**Published:** 2020-09-03

**Authors:** Chien-Chang Ho, Po-Sheng Chang, Hung-Wun Chen, Po-Fu Lee, Yun-Chi Chang, Ching-Yu Tseng, Ping-Ting Lin

**Affiliations:** 1 Department of Physical Education, Fu Jen Catholic University, New Taipei City 242304, Taiwan; 093703@mail.fju.edu.tw (C.-C.H.); 147907@mail.fju.edu.tw (P.-F.L.); d40122003@ym.edu.tw (Y.-C.C.); 015844@mail.fju.edu.tw (C.-Y.T.); 2 Research and Development Center for Physical Education, Health, and Information Technology, College of Education, Fu Jen Catholic University, New Taipei City 242304, Taiwan; 3 Department of Nutrition, Chung Shan Medical University, Taichung 402367, Taiwan; s0746002@gm.csmu.edu.tw (P.-S.C.); s0645019@gm.csmu.edu.tw (H.-W.C.); 4 Graduate Program in Nutrition, Chung Shan Medical University, Taichung 402367, Taiwan; 5 Graduate Institute of Sport Coaching Science, Chinese Culture University, Taipei City 111396, Taiwan; 6 Department of Physical Therapy and Assistive Technology, National Yang-Ming University, Taipei City 112304, Taiwan; 7 Department of Nutrition, Chung Shan Medical University Hospital, Taichung 402367, Taiwan

**Keywords:** ubiquinone supplementation, athletes, glycaemia, antioxidant, sport nutrition

## Abstract

The aim of this study is to investigate the glycemic profile, oxidative stress, and antioxidant capacity in athletes after 12 weeks of ubiquinone supplementation. It was a double-blinded, randomized, parallel, placebo-controlled study. Thirty-one well-trained college athletes were randomly assigned to ubiquinone (300 mg/d, *n* = 17) or placebo group (*n* = 14). The glycemic profile [fasting glucose, glycated hemoglobin (HbA1c), homeostatic model assessment-insulin resistance (HOMA-IR), quantitative insulin sensitivity check index (QUICKI)], plasma and erythrocyte malondialdehyde (MDA), total antioxidant capacity (TAC), and ubiquinone status were measured. After supplementation, the plasma ubiquinone concentration was significantly increased (*p* < 0.05) and the level of erythrocyte MDA was significantly lower in the ubiquinone group than in the placebo group (*p* < 0.01). There was a significant correlation between white blood cell (WBC) ubiquinone and glycemic parameters [HbA1c, *r* = −0.46, *p* < 0.05; HOMA-IR, *r* = −0.67, *p* < 0.01; QUICKI, *r* = 0.67, *p* < 0.01]. In addition, athletes with higher WBC ubiquinone level (≥0.5 nmol/g) showed higher erythrocyte TAC and QUICKI and lower HOMA-IR. In conclusion, we demonstrated that athletes may show a better antioxidant capacity with higher ubiquinone status after 12 weeks of supplementation, which may further improve glycemic control.

## 1. Introduction

Studies have indicated that strenuous exercise may increase the production of reactive oxygen species (ROS) because of an increase in oxygen consumption by the mitochondria and promote oxidative stress in skeletal muscle [1,2,3]. In theory, oral antioxidant supplements could represent a suitable non-invasive tool for preventing or reducing oxidative stress during enduring training [3]. However, it is still controversial whether athletes should take antioxidants [4]. The lack of supportive evidence for recommending antioxidant compounds to athletes may be due to the antioxidant status or conditions of the athletes.

Antioxidants such as ubiquinone (coenzyme Q10) is a popular dietary supplement in athletes. Ubiquinone is a part of the chain of electron transport molecules in mitochondria, which transfers electrons from complexes I and II to complex III and contributes to energy production [5,6,7]. Ubiquinone is a key nutrient for mitochondria because it participates in accepting and transferring electrons and acts as an antioxidant in the mitochondria, particularly mitochondria, which involves many high energy biochemical reactions [8]. A recent systematic review has highlighted that ubiquinone has potential for use as a nutritional supplement to improve exercise capacity or reduce exercise-induced oxidative stress and oxidative stress-related muscle damage and inflammation [9]; however, some inconsistent results may appear because of the dosage of the formulation or timing of the supplementation. In our previous observational study, we found that college athletes exhibit a marginal ubiquinone deficiency [10]. We hypothesize that athletes exhibit a low ubiquinone status owing to their high metabolism requirement. We also note that ubiquinone status seems to play a role in the regulation of glycaemia [10]. Glucose control is important to athletes because they may use it as a fuel for skeletal muscle contraction from glucose regulation during exercise [11,12]. Therefore, we conducted an intervention study to certify the effect of the glycemic panels, oxidative stress, and antioxidant capacity in athletes after 12 weeks of ubiquinone supplementation.

## 2. Materials and Methods

### 2.1. Study Design and Participants’ Inclusion and Exclusion Criteria

The present study was designed as a randomized, double-blinded, placebo-controlled study. College athletes from soccer and Taekwondo teams from Fu Jen Catholic University in Taiwan were enrolled as subjects on March 24, 2018. The athletes were required to train for more than 12 h every week. We excluded participants who were younger than 18 years and individuals who were taking antioxidants or ubiquinone supplements or were under anti-hyperlipidemia or anti-thrombin medications. Informed consent was obtained from each subject. This study was approved by the Institutional Review Board of Fu Jen Catholic University, Taiwan (FJU-IRB C105132) and registered at ClinicalTrials.gov (NCT03321110).

### 2.2. Supplementation

**Figure 1:**
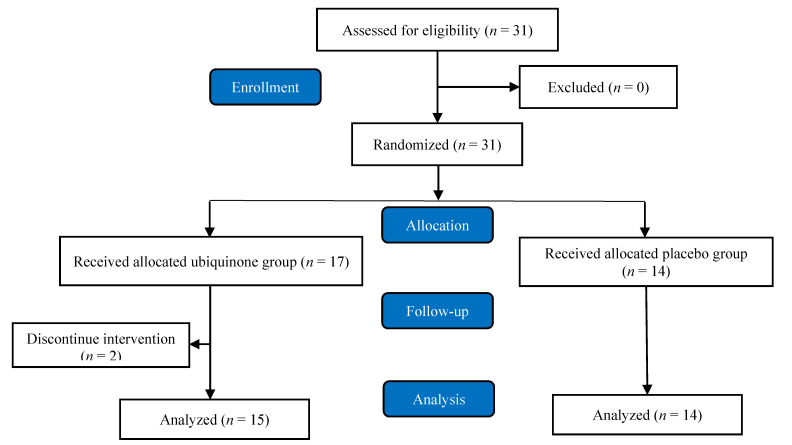
Flow diagram.

The intervention flow diagram is shown in Figure 1. Thirty-one participants were enrolled in this study and randomly assigned to the ubiquinone (*n* = 17) or placebo (*n* = 14) group. The randomization work used the online QuickCalcs random numbers tool (GraphPad QuickCalcs website: https://www.graphpad.com/quickcalcs/randomize1.cfm, San Diego, CA, USA). The ubiquinone and placebo capsules were commercially available preparations (New Health Co., Ltd. Taichung, Taiwan). The subjects were instructed to take a single hard gelatin capsule with 300 mg of ubiquinone powder supplement per day or placebo (a hard gelatin capsule with starch powder per day) after meal with water, and intervention for 12 weeks. With regard to the dosage of ubiquinone at 300 mg/d that we used in the present study was according to our previous clinical studies [13,14]. We found that ubiquinone supplementation at dose of 300 mg/d showed a significant increment in antioxidant enzymes in coronary artery disease patients during statins therapy [13] and hepatocellular carcinoma patients after surgery [14]. Thus, we tried this dosage in athletes to examine the effects of antioxidant capacity after supplementation.

We asked the subjects not to change their diet and lifestyle during the intervention period. Compliance with supplementation was determined as the degree of compliance for each subject according to the number of returned capsules every 6 weeks. Two subjects were lost to follow-up due to transfer or travel abroad. Fifteen and fourteen subjects in the ubiquinone and placebo groups, respectively, completed the supplementation.

### 2.3. Demographic Data Collection and Anthropometric Assessments

The demographic data were collected from questionnaires. With regard to the anthropometric assessments, our department’s research assistants measured subjects’ height, weight, waist circumference, hip circumference, and blood pressure, and the body mass index (BMI) and waist to hip ratio were further calculated. The values for waist and hip circumferences were measured by a tape, the values for body height and weight were measured by a convenient BMI measuring device (InBody BSM370, BioSpace, Seoul, Korea), and blood pressure was measured by a sphygmomanometer (Omron HEM-1000, Kyoto, Japan).

### 2.4. Blood Sample Collection

Fasting blood samples were collected in vacutainer tubes containing ethylenediaminetetraacetic acid anticoagulant (Becton Dickinson, Franklin Lakes, NJ, USA) or sodium fluoride (Sparsh Mediplus, Mumbai, Maharashtra, India), or without anticoagulant as required. Plasma, buffy coat layer, erythrocytes, and serum samples were prepared after centrifugation (3000 rpm, 4 °C, 15 min) and were then stored at −80 °C until analysis. White blood cells (WBC) were obtained from buffy coat layers by using red blood cell lysis buffer [15] for WBC ubiquinone status measurement. Plasma, serum, and erythrocyte samples were used to analyze the following items.

### 2.5. Hematological Measurements

Serum levels of albumin, blood urea nitrogen, creatinine, glutamic oxaloacetic transaminase, glutamic pyruvic transaminase, uric acid, and lipid profiles levels including total cholesterol (TC), triglyceride, low density lipoprotein-cholesterol (LDL-C), and high density lipoprotein-cholesterol (HDL-C) were analyzed by an automated chemistry analyzer (Roche, Cobas 8000, Basel, Switzerland). With regard to glycemic parameter measurements, the levels of glucose and insulin were analyzed by an automated chemistry analyzer (Roche, Cobas 8000, Basel, Switzerland). HbA1c was analyzed by an automated glycated hemoglobin analyzer (Trinity Biotech, Bray, Co., Wicklow, Ireland). The indicators of insulin sensitivity and insulin resistance were further calculated using the following formulas, HOMA-IR (homeostatic model assessment-insulin resistance) = glucose (mmol/L) × insulin (μU/mL)/22.5; and QUICKI (quantitative insulin sensitivity check index) = 1/[log insulin (μU/mL) + log glucose (mg/dL)] [16,17].

### 2.6. Oxidative Stress and Antioxidant Capacity

The level of malondialdehyde (MDA) as an oxidative stress indicator was analyzed in plasma and erythrocytes by reaction of samples with the thiobarbituric acid, followed by heat and extraction with butanol. The spectrophotometer was set at 535 nm to measure the level of MDA [18]. Total antioxidant capacity (TAC) was measured by a Trolox equivalent antioxidant capacity assay in serum and erythrocytes. Free radicals derived from 2,2′-azinobis (3-ethylbenzothiazoline-6-sulfonic acid) were neutralized by the antioxidants in samples, and this reaction could measure the level of TAC by using spectrophotometer set at 730 nm [19].

### 2.7. Ubiquinone Status Measurements

The level of ubiquinone in plasma and WBC was measured by high performance liquid chromatography (HPLC) with an ultraviolet detector as described previously [20]. Briefly, protein in plasma or homogeneous WBC sample was removed by propanol and centrifuged, and then the supernatant with the same ratio of methanol was filtered for analysis by HPLC. The mobile phase was mixed methanol with ethanol. The analysis column (55 mm × 4 mm, 3 μm) used was a LiChroCART^®^RP-18 (Merck, Germany), and the ultraviolet detector was set at 275 nm.

### 2.8. Statistical Analyses

Descriptive data are reported as the means ± standard deviations (median) or percentages. The normal distribution of data was examined by using the Shapiro-Wilk test. Differences of continuous variables between the two groups were assessed by using the Student’s *t*-test or the Mann-Whitney rank sum test. Differences within groups (before and after supplementation) were assessed by using a paired *t*-test or Wilcoxon signed-rank test. For categorical response variables, the differences between the two groups were assessed using the Chi-square test or Fisher’s exact test. To examine the relationship between ubiquinone status, oxidative stress, antioxidant capacity, and glycemic parameters after the supplementation, the Spearman rank order correlation was used. Simple linear regression analyses were further used to examine the association of ubiquinone status, oxidative stress, antioxidant capacity, and glycemic parameters after the supplementation. All analyses in this study were conducted using SigmaPlot software (version 12.0, Systat, San Jose, CA, USA). A *p*-value less than 0.05 was considered statistically significant.

## 3. Results

### 3.1. Baseline Subjects’ Characteristics

The characteristics of the subjects at baseline are shown in Table 1. The age of subjects was between 19 and 20 years. There was no significant difference in age, gender, anthropometric or hematologic data between the two groups at baseline.

### 3.2. Ubiquinone Status, Glycemic Profile, Oxidative Stress, and Antioxidant Capacity

Table 2 shows the levels of ubiquinone, oxidative stress, glucose, and total antioxidant capacity after supplementation. After 12 weeks of supplementation, the levels of plasma ubiquinone, the ratio of plasma ubiquinone to TC, and the level of WBC ubiquinone were significantly increased in the ubiquinone group (*p* < 0.05). In addition, compared with the placebo group, the ubiquinone supplementation group had a significantly higher level of plasma ubiquinone, except for WBC ubiquinone. The level of WBC ubiquinone was increased significantly in the placebo group after the intervention (*p* < 0.05). The level of HbA1c was significantly reduced in both the ubiquinone and placebo groups at week 12 (*p* < 0.05). With regard to oxidative stress and antioxidant capacity, the level of erythrocyte MDA was significantly increased after 12 weeks of supplementation in the placebo group (*p* < 0.05), and the value was also significantly higher than that in the ubiquinone group (*p* < 0.01). Both the levels of serum and the erythrocyte TAC were significantly decreased in the ubiquinone and placebo groups after supplementation (*p* < 0.05).

**Figure 2:**
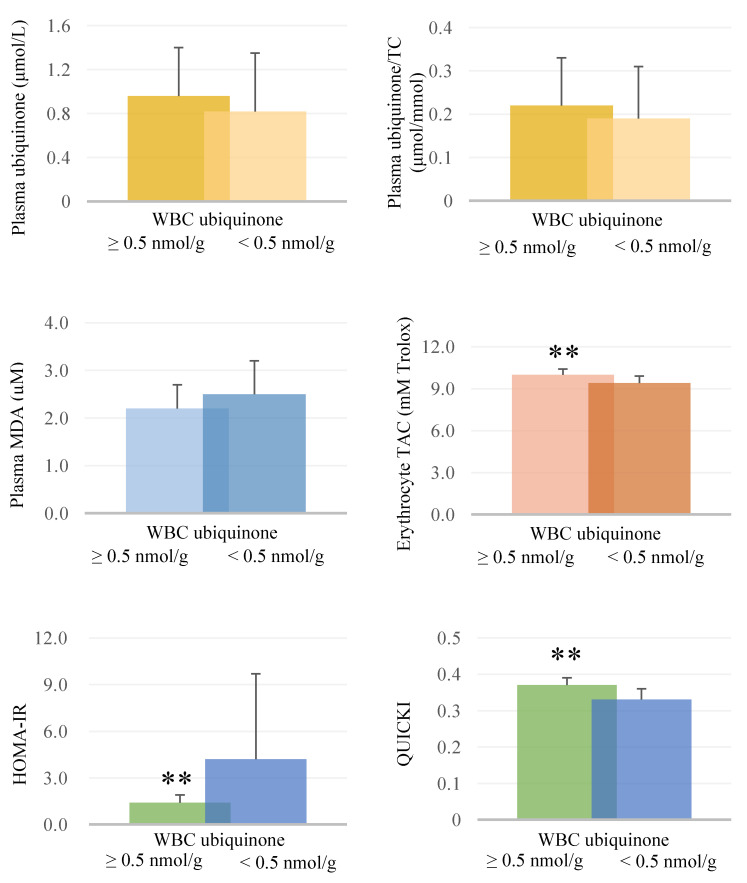
Plasma ubiquinone, oxidative stress, total antioxidant capacity, and glycemic parameters stratified by WBC ubiquinone after supplementation ** *p* < 0.01. HOMA-IR, homeostatic model assessment-insulin resistance; MDA, malondialdehyde; QUICKI, quantitative insulin sensitivity check index; TAC, total antioxidant capacity; TC, total cholesterol; WBC, white blood cell.

We further investigated the levels of plasma ubiquinone, oxidative stress, total antioxidant capacity, and glycemic parameters according to the level of WBC ubiquinone after supplementation (Figure 2). Subjects with a high WBC ubiquinone status (≥0.5 nmol/g) had significantly higher levels of erythrocyte TAC and QUICKI than those in the low WBC ubiquinone group (*p* < 0.01). The HOMA-IR value was significantly lower in the high WBC ubiquinone status than in the low WBC ubiquinone group (*p* < 0.01).

### 3.3. Correlations between Ubiquinone Status and Oxidative Stress, Total Antioxidant Capacity, and Glycemic Parameters after Supplementation

Table 3 shows the correlations between ubiquinone status and oxidative stress, total antioxidant capacity, and glycemic parameters after supplementation. Plasma or WBC ubiquinone status was significantly negatively correlated with plasma and erythrocyte MDA levels (*p* < 0.05). WBC ubiquinone level was significantly positively correlated with serum TAC and QUICKI (*p* < 0.01); and negatively correlated with glycemic parameters such as HbA1c and HOMA-IR (*p* < 0.01) after 12 weeks of supplementation. We further examined the effects of ubiquinone status on oxidative stress, antioxidant capacity, and glycemic parameters. The results show that an increment of 1 μmol/L plasma ubiquinone status may lower the erythrocyte MDA levels by 0.38 nmol/mg protein (*β* = 0.38, *p* = 0.03); an increment of 1 μmol/mmol plasma ubiquinone/TC status may significantly lower the erythrocyte MDA levels by 1.66 nmol/mg protein (*β* = 1.66, *p* = 0.03) and slightly plasma MDA levels by 1.92 μM (*β* = 1.92, *p* = 0.08). An increment of 1 nmol/g WBC ubiquinone level may significantly increase the QUICKI by 0.02 (*β* = 0.02, *p* = 0.03) and slightly increase the level of serum TAC by 0.31 mM Trolox (*β* = 0.31, *p* = 0.08).

### 3.4. Correlations between Oxidative Stress, Antioxidant Capacity, and Glycemic Parameters after Supplementation

Table 4 shows data on the correlations between oxidative stress, antioxidant capacity, and glycemic parameters after supplementation. There was a positive correlation between erythrocyte MDA and HbA1c (*p* < 0.05). In addition, serum TAC was significantly correlated with HOMA-IR and QUICKI after supplementation (*p* < 0.05). Further to examine the effects of oxidative stress and antioxidant capacity on glycemic parameters after supplementation. The results show that a decrease of 1 nmol/mg protein erythrocyte MDA level may significantly lower HbA1c by 0.27% (*β* = 0.27, *p* < 0.05) and an increment of 1 mM Trolox serum TAC level may slightly increase the QUICKI by 0.02 (*β* = 0.02, *p* = 0.06).

## 4. Discussion

Research on the use of ubiquinone as an antioxidant supplement to reduce increased oxidative stress during exercise remains controversial. Current evidences concluded that ubiquinone supplementation might show improvements in systemic markers of oxidative stress or antioxidant enzymes after supplementation [4]. In the present study, we failed to detect a significant reduction in the level of oxidative stress (MDA) after supplementation; however, it is worth noting that in athletes in the placebo group, the level of erythrocyte MDA was significantly increased after 12 weeks of intervention (Table 2). Although the antioxidant capacity was decreased significantly in both groups, ubiquinone status was significantly negatively correlated with oxidative stress and positively correlated with antioxidant capacity after the intervention (Table 3). Emami et al. recently reported a series of data for using oral ubiquinone supplementation (300 mg/d) along with a precooling strategy in elite swimmers and found that oral ubiquinone supplementation prevented adverse changes in oxidative stress, antioxidant enzymes, and inflammatory mediators during intense swimming training [21,22,23]. Supplementation for ubiquinone with 300 mg/d seems to show that a plateau in ubiquinone status may further alleviate exercise-induced muscle injury and muscle fatigue [24,25] and enhance peak power production [26]. In addition to a dosage of ubiquinone supplementation at 300 mg/d, two previous studies conducted by Sarmiento et al. [27] and Orlando et al. [28], they tried to using a dosage at 200 mg/d of ubiquinol supplementation in the healthy adults with strenuous exercise and intense exercise young athletes, respectively. Both studies found that ubiquinol supplementation can minimize exercise-induced depletion and enhanced plasma and cellular antioxidant capacity [27,28]. The ubiquinone supplementation that we used in the present study is an oxidized form of ubiquinone. After an oral intake of ubiquinone supplement, about 95% of ubiquinone may covert to ubiquinol (a reduced form of ubiquinone) to play a free radicals scavenger, and the ratio may not be affected by the oral ingestion of ubiquinone or ubiquinol [29,30]. An investigated study recently points out that the bioavailability of ubiquinone or ubiquinol supplements may depend on its formulations and physiological status of the individual to affect the efficiency of the supplementation [31]. A single dose of 300 mg/d ubiquinone supplementation that we used in the present study has successfully increased plasma ubiquinone status in athletes. In view of the importance of oxidative stress associated with the intensity of exercise, it could be considered to assess the antioxidant effect of oral supplementation, such as ubiquinone.

In the present study, we measured the WBC ubiquinone as an indicator of the ubiquinone status in the mitochondria. Exercise training modifies the function of skeletal muscle mitochondria to enhance mitochondrial ATP production and substrate metabolism [32]. Both groups of athletes in the present study were observed during the competition season and received regular exercise training, which may have contributed to an increasing level of WBC ubiquinone in both groups at week 12 (Table 2). As presented in Figure 2, we found that a WBC ubiquinone status higher than 0.5 nmol/g showed a better antioxidant capacity than did low WBC ubiquinone status. A WBC ubiquinone level ≥ 0.5 nmol/g is equivalent to a plasma ubiquinone of 1.0 μmol/L or a plasma ubiquinone/total cholesterol level of 0.2 μmol/mmol. These values for ubiquinone status must be achieved by dietary supplementation not exercise alone (Table 2). Two latest studies recently revealed the beneficial effect of ubiquinol supplementation at doses of 200–300 mg/d in athletes, the results showed that ubiquinone not only could modulate inflammatory signaling, such as decreased the expression of pro-inflammatory cytokines or increased anti-inflammatory cytokines [33] but also could mitigate tissue damage and alleviate fatigue [25] in athletes during strenuous exercise. Physical activity has cardiovascular protection because of regulating the β adrenergic system [34]. However, the exercise intensity is positively correlated with the production of inflammatory cytokines [35]. Thus, it is worth trying a dietary supplement in exercise training because it could modulate the inflammatory cytokines signaling to reduce the chronic inflammation, and the supplement could exert remarkable anti-inflammation effect than diet or exercise alone [35]. In the present study, we have measured the level of an inflammatory indicator (high sensitivity C-reactive protein) during the intervention, but we did not found an anti-inflammation effect of ubiquinone supplementation in these subjects (data not shown). We suspected that because our subjects are healthy adults, they did not suffer from a high inflammation status during the intervention. Further study could recruit athletes who suffer from a high inflammation state during exercise to clarify the anti-inflammatory effect of ubiquinone supplementation. Since acute and strenuous exercise may result in an imbalance in oxidative stress and antioxidant status and ubiquinone plays a role as an antioxidant in mitochondria by scavenging free radicals directly [3], we suggest that athletes could use an ubiquinone supplement as dietary supplementation to improve their antioxidant capacity by increasing the ubiquinone status during exercise training.

Exercise can improve insulin sensitivity and thereby alter skeletal muscle glucose transport and maintain blood glucose homeostasis, especially for patients with metabolic disease [36], but may not always have the same effect in athletes due to excessive production of ROS during exercise training [37,38,39]. In this study, we did not detect a significant reduction in glucose parameters in the ubiquinone group after supplementation; however, we found that athletes with higher WBC ubiquinone level (≥0.5 nmol/g) showed improved glycemic control (Table 3 and Figure 2), which might be related to an increase in antioxidant capacity (Table 4). These results support the inference of our previous observational study [10]. In fact, ubiquinone supplementation is beneficial for glycemic control, as demonstrated in many clinical studies [40,41,42]. It has been proposed that ubiquinone can improve insulin sensitivity through modulation of the insulin receptor and glucose transporters (GLUT4) [43] and by increasing antioxidant capacity [42] after ubiquinone supplementation. Athletes may face glycemic control during exercise training and workload [10,44,45]. Thus, our results support that athletes may reduce oxidative stress and increase antioxidant activity for adjuvant modulation of glycemic control by using ubiquinone supplementation during exercise training. Recently, the mitochondria-targeted antioxidant Mitoquinone (MitoQ) has been developed. MitoQ is an enhanced form of ubiquinone which is more directly absorbed into the mitochondria than standard ubiquinone supplements [46]. A study has tried to explore the antioxidant effect of MitoQ supplementation (20 mg/d) in healthy young males with high-intensity intermittent exercise [47]. The researchers have found MitoQ could protect genome stability, against DNA damage in exercising humans; however, this protective effect was only unmasked by exercise [47]. An adequate physical exercise is able to enhance endogenous antioxidant defense system [48]; on the other hand, exhaustive exercise may exacerbate metabolic disorder and ROS production in skeletal muscle [49]. We support the researchers noted that it is necessary to understand the physiological status of individuals as well as the intensity of exercise, the types of exercise (aerobic or anaerobic), or the duration and formulations for antioxidants when appraising the efficacy of antioxidants in athletes [47,48,49,50].

Age plays a key factor that can affect a person’s oxidative stress. The theory for oxidative stress of aging is the accumulation of reactive oxygen and nitrogen species that induces cells or organs damages [51]. In the present study, our subjects were young college athletes aged between 19 and 20 years, and there was no significant difference in age between ubiquinone and placebo groups at baseline (Table 1). In addition, we have examined the correlations between age and ubiquinone status, and oxidative stress markers (plasma and erythrocyte MDA) before and after intervention by using linear regression analyses. There was no significant correlations between age and ubiquinone status, and oxidative stress before and after intervention (data not shown). Thus, age may not be a confounding factor in the present study.

The strength of this investigation is that it is the first study to clarify the causal relationship between ubiquinone status and antioxidant capacity and glycemic control after ubiquinone supplementation. The present study also has some limitations that should be mentioned. First of all, the sample size of this study was small and it combined two events of sports for soccer and taekwondo. However, both of these athletes belonged to explosive lower limbs exercise training and the subjects’ characteristics in soccer and taekwondo were not significantly different between the ubiquinone and placebo groups. As a result, we combined these two sports in order to increase the sample size. Second, the duration of the ubiquinone supplementation was not long enough. In fact, most of the ubiquinone interventional studies in athletes were administered for a short-term (4–8 weeks) [9]; the present study lasted for 12 weeks. Large-scale and long-term interventions should be further conducted to verify the results of this study. Third, diet reports bias in the present study. As a matter of fact, we have instructed the subjects to perform the 3-days diet records before blood sample collection during the study, and also asked them not to change the diet through the intervention. We have analyzed the dietary intake by the Nutritionist Professional software package (version enhancement plus 3, E-Kitchen Business Corp., Taichung, Taiwan) and found that there was no significant difference in daily energy, carbohydrate, protein, and fat intakes between ubiquinone and placebo groups at baseline and after intervention (data not shown). The previous studies indicated that ubiquinone can be obtained from foods; it is mostly found in the meats such as heart, kidney, and liver, and meats processed foods. However, the average dietary intake of ubiquinone from foods is only 3 to 6 mg, then following circulation in the plasma and absorption in the intestine [30,52]. In addition, we noted that the plasma ubiquinone concentration doubled after ubiquinone intervention (Table 2, 0.57 ± 0.18 μmol/L increased to 1.14 ± 0.54 μmol/L, *p* < 0.01), but there was no significant change in the placebo group after intervention (Table 2, 0.58 ± 0.16 μmol/L increased to 0.60 ± 0.20 μmol/L, *p* = 0.55). Therefore, the diet may not be a key factor to contribute an increment in ubiquinone status in the present study. However, we found that our subjects may under-reporting their dietary intake because of low daily energy intake (about 1500 kcal/d). We found that most of the subjects did not report the meal of breakfast. This dietary reporting bias of the subjects is also a limitation of this study. With regard to the dietary control during the intervention, we recommend using a combination of 24-h dietary recall questionnaire and dietary records to more accurately monitor the food intake of the subjects.

## 5. Conclusions

Athletes may develop high oxidative stress from exercise training. In the present study, we found that ubiquinone supplementation may yield an increase of ubiquinone status and further increase antioxidant capacity, which benefits glycemic control in athletes.

## Figures and Tables

**Table 1:** Characteristics of subjects at baseline.

Characteristics	Ubiquinone (*n* = 15)	Placebo (*n* = 14)	*p* Value
Age (years)	19.9 ± 1.3 (20.0) ^1^	19.6 ± 1.3 (19.0)	0.56
Sports			0.87
Soccer (*n*, %)	9 (60.0%)	7 (50.0%)	
Males/Females (*n*)	4/5	5/2	0.36
Taekwondo (*n*, %)	6 (40.0%)	7 (50.0%)	
Males/Females (*n*)	4/2	7/1	0.56
Body mass index (kg/m^2^)	22.4 ± 1.8 (22.8)	22.1 ± 2.8 (22.2)	0.76
Waist-hip ratio	0.79 ± 0.04 (0.79)	0.79 ± 0.05 (0.78)	0.76
Systolic blood pressure (mmHg)	116.4 ± 13.6 (114.0)	125.2 ± 13.9 (120.0)	0.10
Diastolic blood pressure (mmHg)	67.9 ± 9.3 (65.0)	70.4 ± 7.4 (70.0)	0.45
Hematology			
Albumin (g/L)	49.9 ± 3.0 (51.0)	50.1 ± 2.6 (49.0)	0.84
Blood urea nitrogen (mmol/L)	5.2 ± 1.2 (5.4)	5.1 ± 1.3 (5.0)	0.93
Creatinine (μmol/L)	68.6 ± 11.4 (71.7)	84.0 ± 14.1 (85.7)	0.39
GOT (U/L)	20.5 ± 6.9 (21.0)	20.0 ± 3.9 (19.0)	0.83
GPT (U/L)	17.6 ± 11.2 (13.0)	15.9 ± 5.0 (16.0)	0.65
Uric acid (μmol/L)	350.9 ± 77.3 (380.7)	374.8 ± 65.4 (398.5)	0.43
TC (mmol/L)	4.4 ± 0.7 (4.7)	4.4 ± 0.9 (4.2)	1.00
Triglyceride (mmol/L)	0.69 ± 0.30 (0.55)	0.84 ± 0.81 (0.62)	0.97
LDL-C (mmol/L)	2.4 ± 0.6 (2.5)	2.6 ± 0.8 (2.4)	0.46
HDL-C (mmol/L)	1.9 ± 0.3 (1.9)	1.6 ± 0.4 (1.6)	0.07

^1^ mean ± SD (median). GOT, glutamic oxaloacetic transaminase; GPT, glutamic pyruvic transaminase; HDL-C, high density lipoprotein-cholesterol; LDL-C, low density lipoprotein-cholesterol; TC, Total cholesterol.

**Table 2:** Ubiquinone status, glycemic profile, oxidative stress, and total antioxidant capacity after supplementation.

Parameters	Ubiquinone (*n* = 15)	Placebo (*n* = 14)	*p* Value
Ubiquinone status			
Plasma ubiquinone (μmol/L)-Baseline	0.57 ± 0.18 (0.53) ^1^	0.58 ± 0.16 (0.54)	0.92
Plasma ubiquinone (μmol/L)-12 weeks	1.14 ± 0.54 (1.06) *	0.60 ± 0.20 (0.60)	<0.01
Plasma ubiquinone/TC (μmol/mmol)-Baseline	0.13 ± 0.03 (0.13)	0.13 ± 0.04 (0.13)	0.66
Plasma ubiquinone/TC (μmol/mmol)-12 weeks	0.27 ± 0.12 (0.26) *	0.14 ± 0.04 (0.14)	<0.01
WBC ubiquinone (nmol/g)-Baseline	0.42 ± 0.32 (0.42)	0.31 ± 0.21 (0.30)	0.28
WBC ubiquinone (nmol/g)-12 weeks	0.75 ± 0.71 (0.48) *	0.55 ± 0.43 (0.49) *	0.45
Glycemic profile			
Fasting glucose (mmol/L)-Baseline	4.7 ± 0.5 (4.7)	5.0 ± 0.3 (4.9)	0.10
Fasting glucose (mmol/L)-12 weeks	5.0 ± 1.0 (4.8)	4.7 ± 0.4 (4.7)	0.46
HbA1c (%)-Baseline	5.4 ± 0.4 (5.4)	5.5 ± 0.2 (5.6)	0.38
HbA1c (%)-12 weeks	5.3 ± 0.4 (5.3) ^†^	5.4 ± 0.3 (5.4) *	0.55
Oxidative stress			
Plasma MDA (μM)-Baseline	2.5 ± 0.5 (2.5)	2.3 ± 0.4 (2.4)	0.31
Plasma MDA (μM)-12 weeks	2.4 ± 0.7 (2.4)	2.4 ± 0.7 (2.3)	1.00
Erythrocyte MDA (nmol/mg protein)-Baseline	3.7 ± 0.3 (3.7)	3.8 ± 0.3 (3.8)	0.34
Erythrocyte MDA (nmol/mg protein)-12 weeks	3.6 ± 0.3 (3.6)	4.1 ± 0.5 (4.0) *	<0.01
Antioxidant capacity			
Serum TAC (mM Trolox)-Baseline	5.7 ± 0.4 (5.8)	5.7 ± 0.3 (5.8)	0.53
Serum TAC (mM Trolox)-12 weeks	5.2 ± 0.4 (5.2) *	5.2 ± 0.2 (5.2) *	0.88
Erythrocyte TAC (mM Trolox)-Baseline	10.4 ± 0.5 (10.5)	10.7 ± 0.6 (10.6)	0.16
Erythrocyte TAC (mM Trolox)-12 weeks	9.7 ± 0.5 (9.8) *	9.7 ± 0.6 (9.8) *	0.90

^1^ mean ± SD (median). *^,†^ Values were compared within groups, * *p* < 0.05, ^†^ *p* = 0.06. HbA1_C_, glycated hemoglobin; MDA, malondialdehyde; TAC, total antioxidant capacity; TC, total cholesterol; WBC, white blood cell.

**Table 3:** Correlations between ubiquinone status and oxidative stress, antioxidant capacity, and glycemic parameters after supplementation.

Parameters	Plasma Ubiquinone (μmol/L)	Plasma Ubiquinone/TC (μmol/mmol)	WBC Ubiquinone (nmol/g)
	Correlation Coefficients ^1^		
Oxidative stress			
Plasma MDA (μM)	−0.35 ^†^	−0.40 *	−0.15
Erythrocyte MDA (nmol/mg protein)	−0.46 *	−0.43 *	−0.44 **
Antioxidant capacity			
Serum TAC (mM Trolox)	0.27	0.23	0.57 **
Erythrocyte TAC (mM Trolox)	−0.09	−0.10	0.19
Glycemic parameters			
Fasting glucose (mmol/L)	−0.02	0.01	−0.09
HbA1c (%)	−0.23	−0.19	−0.46 *
HOMA-IR	−0.15	−0.19	−0.67 **
QUICKI	0.15	0.19	0.67 **

^1^ Spearman rank order correlation coefficients. * *p* < 0.05, ** *p* < 0.01, ^†^ *p* = 0.06. HbA1_C_, glycated hemoglobin; HOMA-IR, homeostatic model assessment-insulin resistance; MDA, malondialdehyde; QUICKI, quantitative insulin sensitivity check index; TAC, total antioxidant capacity; TC, total cholesterol; WBC, white blood cell.

**Table 4:** Correlations between oxidative stress, antioxidant capacity, and glycemic parameters after supplementation.

Parameters	Plasma MDA (μM)	Erythrocyte MDA (nmol/mg Protein)	Serum TAC (mM Trolox)	Erythrocyte TAC (mM Trolox)
	Correlation Coefficients ^1^			
Fasting glucose (mmol/L)	0.06	−0.03	−0.05	0.18
HbA1c (%)	0.28	0.40 *	−0.25	−0.14
HOMA-IR	0.22	0.13	−0.44 *	−0.04
QUICKI	−0.22	−0.13	0.44 *	0.04

^1^ Spearman rank order correlation coefficients. * *p* < 0.05. HbA1_C_, glycated hemoglobin; HOMA-IR, homeostatic model assessment-insulin resistance; MDA, malondialdehyde; QUICKI, quantitative insulin sensitivity check index; TAC, total antioxidant capacity.

## Abbreviations

BMI, body mass index; HbA1_C_, glycated hemoglobin; HDL-C, high density lipoprotein-cholesterol; HOMA-IR, homeostatic model assessment-insulin resistance; LDL-C, low density lipoprotein-cholesterol; MDA, malondialdehyde; MitoQ, Mitoquinone; QUICKI, quantitative insulin sensitivity check index; ROS, reactive oxygen species; TAC, total antioxidant capacity; TC, total cholesterol; WBC, white blood cell.
